# Precise diagnosis of three top cancers using dbGaP data

**DOI:** 10.1038/s41598-020-80832-x

**Published:** 2021-01-12

**Authors:** Xu-Qing Liu, Xin-Sheng Liu, Jian-Ying Rong, Feng Gao, Yan-Dong Wu, Chun-Hua Deng, Hong-Yan Jiang, Xiao-Feng Li, Ye-Qin Chen, Zhi-Guo Zhao, Yu-Ting Liu, Hai-Wen Chen, Jun-Liang Li, Yu Huang, Cheng-Yao Ji, Wen-Wen Liu, Xiao-Hu Luo, Li-Li Xiao

**Affiliations:** 1 Huaiyin Institute of Technology, Huaian, 223003 China; 2 Nanjing University of Aeronautics and Astronautics, Nanjing, 210016 China; 3 Jiangsu Vocational College of Electronics and Information, Huaian, 223003 China; 4 Georgia State University, Atlanta, 30303 USA

**Keywords:** Breast cancer, Lung cancer, Prostate cancer, Breast cancer, Lung cancer, Prostate cancer

## Abstract

The challenge of decoding information about complex diseases hidden in huge number of single nucleotide polymorphism (SNP) genotypes is undertaken based on five dbGaP studies. Current genome-wide association studies have successfully identified many high-risk SNPs associated with diseases, but precise diagnostic models for complex diseases by these or more other SNP genotypes are still unavailable in the literature. We report that lung cancer, breast cancer and prostate cancer as the first three top cancers worldwide can be predicted precisely via 240–370 SNPs with accuracy up to 99% according to leave-one-out and 10-fold cross-validation. Our findings (1) confirm an early guess of Dr. Mitchell H. Gail that about 300 SNPs are needed to improve risk forecasts for breast cancer, (2) reveal an incredible fact that SNP genotypes may contain almost all information that one wants to know, and (3) show a hopeful possibility that complex diseases can be precisely diagnosed by means of SNP genotypes without using phenotypical features. In short words, information hidden in SNP genotypes can be extracted in efficient ways to make precise diagnoses for complex diseases.

## Introduction

High-throughput sequencing technology helps us get more and more molecular data, but also poses challenges on how to use these rich resources efficiently^1^. Among these challenges, it is of great practical significance to find methods of diagnosing complex diseases precisely based on single nucleotide polymorphism (SNP) genotypes^2,3^. This challenge has become a shackle to current genome-wide association (GWA) studies, and now it may be the time to break it such that moving beyond the initial steps of GWA studies^4^ will be no longer a hard work in the near future.

According to the global cancer statistics 2018^5^, lung cancer^6–8^, breast cancer^9–11^ and prostate cancer^12–15^ are still the first three top cancers around the world (30.3% of the total cases and 28.8% of the total cancer deaths), so we start exploration from these three cancers. Our method can be extended to more other complex diseases, and may be expected to serve for personalized diagnosis and even precise medicine^16,17^. If so, combination of precise diagnostic models with those important insights known in GWA studies shall play a substantial role in further promoting GWA studies and even in improving human health comprehensively^4^.

**Figure 1:**
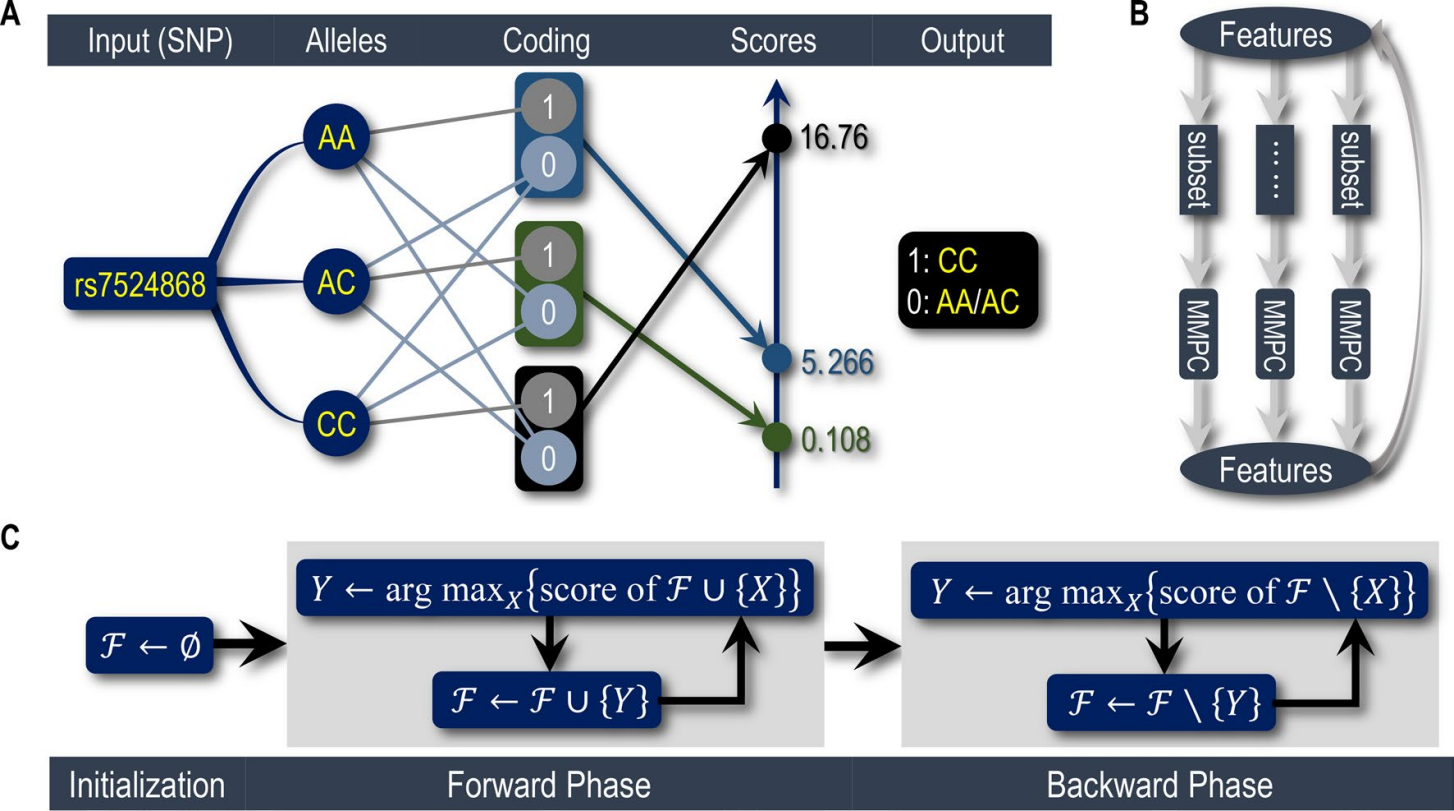
Main idea of building precise diagnostic models. (**A**) An illustration on how Snp2Bin works, taking the SNP, rs7524868 of phs000634, for example. Here, the score of a coding-scheme is defined as the $\chi ^2$-statistic of the corresponding contingency table. (**B**) Schematic of IterMMPC. (**C**) Pseudocode for OptNBC, which consists of forward and backward phases.

Five dbGaP studies (containing six datasets in total) related to these three cancers are studied, with the following accession numbers: phs000634.v1.p1, phs000753.v1.p1^7^, phs000147.v3.p1^9,10^, phs000517.v3.p1 and phs000306.v4.p1. Given a dataset, we first use Snp2Bin (Fig. 1A and Algorithm S1; a key procedure) to transform SNPs into 2-value variables; Then, apply IterMMPC (Fig. 1B and Algorithm S2) to reduce attributes; Finally, employ OptNBC (Fig. 1C and Algorithm S3) to get the optimal features for naive Bayes classifier (NBC^18,19^).

## Results

### Classifications by means of OptNBC-based models

**Figure 2:**
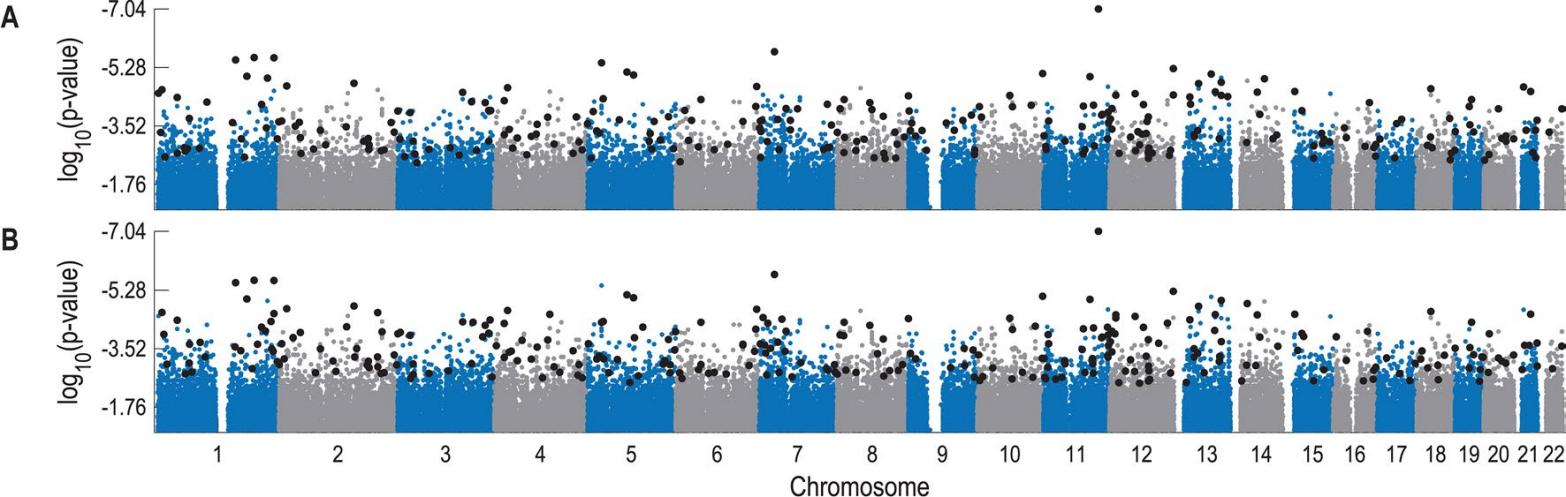
$\mathrm {Log}_{10}(p\,\hbox {value})$ of SNPs associated with lung cancer risk based on the data from phs000634. Small blue or gray dots denote all of the 656,891 SNPs with $\mathrm {log}_{10}(p\,\hbox {value})$ not larger than $-1$; large black dots denote the SNPs used in our NBC models. (**A**) Result of ${\texttt {NBC}_{\texttt {634}}^{\texttt {(1)}}}$. (**B**) Result of ${\texttt {NBC}_{\texttt {634}}^{\texttt {(2)}}}$.

The lung cancer study, , consists of 946 cases and 1052 controls, involving 656,891 SNPs. Following the above procedures, we first use Snp2Bin to transform these SNPs into 656,891 binary variables, then apply IterMMPC to reduce attributes and obtain a 3274-variable subset, and finally employ OptNBC to get a 268-feature NBC (Fig. 2A and Fig. S1A).

**Figure 3:**
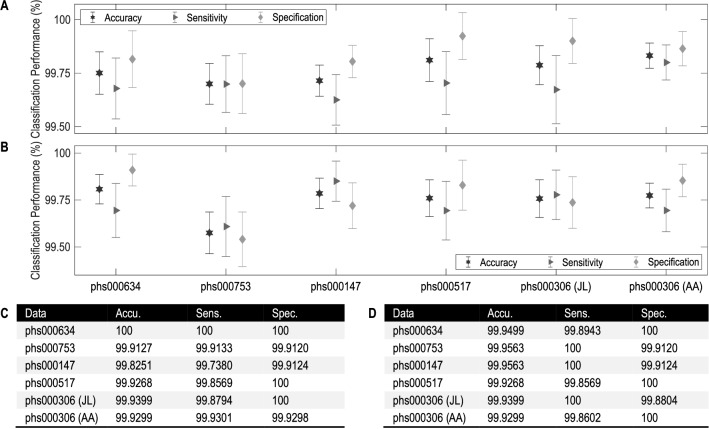
Classification performance of NBCs over the six datasets from five dbGaP studies. (**A**) Accuracy, sensitivity and specification of ${\texttt {NBC}_{\texttt {634}}^{\texttt {(1)}}}$, ${\texttt {NBC}_{\texttt {753}}^{\texttt {(1)}}}$, ${\texttt {NBC}_{\texttt {147}}^{\texttt {(1)}}}$, ${\texttt {NBC}_{\texttt {517}}^{\texttt {(1)}}}$, ${\texttt {NBC}_{\texttt {306}\hbox {-}{} \texttt {JL}}^{\texttt {(1)}}}$ and ${\texttt {NBC}_{\texttt {306}\hbox {-}{} \texttt {AA}}^{\texttt {(1)}}}$ according to 10 fold cross-validation, where the error bars are in form of “mean±std” computed by repeatedly performing 10-fold cross-validation for 100 times. (**B**) Performance of ${\texttt {NBC}_{\texttt {634}}^{\texttt {(2)}}}$, ${\texttt {NBC}_{\texttt {753}}^{\texttt {(2)}}}$, ${\texttt {NBC}_{\texttt {147}}^{\texttt {(2)}}}$, ${\texttt {NBC}_{\texttt {517}}^{\texttt {(2)}}}$, ${\texttt {NBC}_{\texttt {306}\hbox {-}{} \texttt {JL}}^{\texttt {(2)}}}$ and ${\texttt {NBC}_{\texttt {306}\hbox {-}{} \texttt {AA}}^{\texttt {(2)}}}$ according to 10-fold cross-validation. (**C**) Accuracy (accu.; %), sensitivity (sens.; %) and specification (spec.; %) of each ${\texttt {NBC}_{\texttt {study}}^{\texttt {(1)}}}$ according to leave-one-out. (**D**) Performance of each ${\texttt {NBC}_{\texttt {study}}^{\texttt {(2)}}}$ according to leave-one-out.

**Figure 4:**
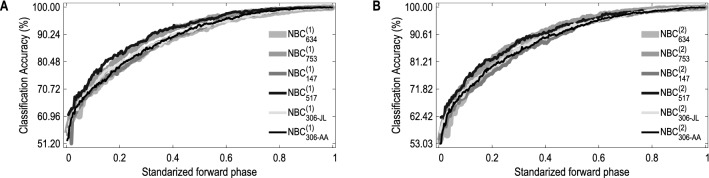
Classification accuracy of NBCs versus the standardized forward phase. Here, the accuracy is that of NBCs in the modeling process, computed according to leave-one-out; the forward phase of OptNBC or SubOptNBC is “standardized” in the sense that “0” and “1” stand for its first and last steps, respectively. (**A**) Results based on OptNBC. (**B**) Results based on SubOptNBC.

For convenience, denote this NBC model by ${\texttt {NBC}_{\texttt {634}}^{\texttt {(1)}}}$. Its classification accuracy according to leave-one-out is 100% (Figs. 3C, 4A). The other lung cancer study, phs000753, consists of 1153 cases and 1137 controls, involving 317,498 SNPs. For this dataset, we get a 1298-variable subset and then a 343-feature NBC (Figs. S1B and S3A), denoting it by ${\texttt {NBC}_{\texttt {753}}^{\texttt {(1)}}}$. Its classification accuracy according to leave-one-out is 99.91% (Figs. 3C, 4A).

The two breast cancer studies, phs000147 and phs000517, consist of 1145/699 cases and 1142/667 controls, involving 546,646 and 1,288,157 SNPs, respectively. For phs000147, we get a 4128-variable subset and then a 318-feature NBC; for phs000517, we get a 1863-variable subset and then a 255-feature NBC. Denote the two NBCs by ${\texttt {NBC}_{\texttt {147}}^{\texttt {(1)}}}$ and ${\texttt {NBC}_{\texttt {517}}^{\texttt {(1)}}}$, respectively (Figs. S1C and S3B; S1D and S5A). These two NBCs perform classification with accuracy 99.83% and 99.93% according to leave-one-out (Figs. 3C, 4A).

The prostate cancer study, phs000306, is divided into two datasets: one is for Japanese and Latinos (JL) containing 829 cases and 836 controls, the other is for African Americans (AA) containing 1431 cases and 1424 controls. JL and AA contain 657,366 and 1,199,187 SNPs, respectively. For JL, we get a 3919-variable subset and then a 242-feature NBC; for AA, we get a 24,457-variable subset and then a 352-feature NBC. Denote these two NBCs by ${\texttt {NBC}_{\texttt {306}\hbox {-}{} \texttt {JL}}^{\texttt {(1)}}}$ and ${\texttt {NBC}_{\texttt {306}\hbox {-}{} \texttt {AA}}^{\texttt {(1)}}}$, respectively (Figs. S1E and S3C; S1F and S3D). The classification accuracy of ${\texttt {NBC}_{\texttt {306}\hbox {-}{} \texttt {JL}}^{\texttt {(1)}}}$ according to leave-one-out is 99.94%, and that of ${\texttt {NBC}_{\texttt {306}\hbox {-}{} \texttt {AA}}^{\texttt {(1)}}}$ is 99.93% (Figs. 3C, 4A). Note that the SNPs selected for JL are almost completely different from that for AA. This indicates diagnosis of prostate cancer based on SNP genotypes depends on ethnicity^20,21^, showing the same conclusion as Yücebaş and Son^14^ concluded that ethnicity is the most important attribute.

Besides the classification accuracy, we also compute Matthews correlation coefficients (MCCs^22^) to measure the performance (Table S7E), each of which is larger than 0.99.

To further evaluate the classification performance of the above six NBCs, for each dataset we repeatedly perform 10-fold cross-validation for 100 times by randomly dividing all data points into 10 subsets and then performing a procedure similar to leave-one-out. The values of accuracy, sensitivity and specification are used to get corresponding error bars (Fig. 3A). As seen, the predictive performance of each NBC is very desirable. The MCCs show the same conclusion as above (Table S7F).

### Classifications by means of **SubOptNBC**-based models

Although classification performance of each NBC is satisfactory according to leave-one-out (Fig. 3C) and 10-fold cross-validation (Fig. 3A), here are two problems to be solved: (a) There are a few incorrect diagnoses (e.g., the 318th instance diagnosed by ${\texttt {NBC}_{\texttt {753}}^{\texttt {(1)}}}$; Table 2C), for which what can we do? (b) There are some doubtful diagnoses (with posterior probability of being diagnosed as “positive” approximating 0.5; e.g., the 811st instance diagnosed by ${\texttt {NBC}_{\texttt {634}}^{\texttt {(1)}}}$; Table 1A), for which what can we do?

To address these two issues, a simple solution is to look for an alternative NBC for each ${\texttt {NBC}_{\texttt {study}}^{\texttt {(1)}}}$, written as ${\texttt {NBC}_{\texttt {study}}^{\texttt {(2)}}}$, which should also perform desirably, and then complement them with each other according to some rule.

Following this idea, we modify OptNBC slightly to obtain the SubOptNBC algorithm (involved in Algorithm S3). Substituting SubOptNBC for OptNBC in the process of building models for the six datasets, we get a 290-feature NBC for phs000634 denoted by ${\texttt {NBC}_{\texttt {634}}^{\texttt {(2)}}}$, a 329-feature NBC for phs000753 denoted by ${\texttt {NBC}_{\texttt {753}}^{\texttt {(2)}}}$, a 307-feature NBC for phs000147 denoted by ${\texttt {NBC}_{\texttt {147}}^{\texttt {(2)}}}$, a 249-feature NBC for phs000517 denoted by ${\texttt {NBC}_{\texttt {517}}^{\texttt {(2)}}}$, a 258-feature NBC for JL of phs000306 denoted by ${\texttt {NBC}_{\texttt {306}\hbox {-}{} \texttt {JL}}^{\texttt {(2)}}}$, and a 367-feature NBC for AA of phs000306 denoted by ${\texttt {NBC}_{\texttt {306}\hbox {-}{} \texttt {AA}}^{\texttt {(2)}}}$ (Figs. S2, S4 and S5B). These six NBCs perform classification with accuracy 99.95%, 99.96%, 99.96%, 99.93%, 99.94% and 99.93% according to leave-one-out (Figs. 3D, 4B) and not less than 99% according to 10-fold cross-validation (Fig. 3B), also performing well enough. Their MCCs show similar results (Table S7E).

**Table 1:** Performance of remedying procedures for all possible situations of phs000634.

Instance no.	True status	${\texttt {NBC}_{\texttt {634}}^{\texttt {(1)}}}$	${\texttt {NBC}_{\texttt {634}}^{\texttt {(2)}}}$	Conclusion	Instance no.	True status	${\texttt {NBC}_{\texttt {634}}^{\texttt {(2)}}}$	${\texttt {NBC}_{\texttt {634}}^{\texttt {(1)}}}$	Conclusion
(A)					(B)				
118	Case	0.5132	0.6442	Improved	29	Case	0.4896	0.6215	Corrected
811	Case	0.5005	0.9954	Improved	39	Case	0.5495	0.9332	Improved
1024	Case	0.5225	0.9034	Improved	375	Case	0.5290	0.9450	Improved
1077	Control	0.4590	0.2712	Improved	435	Control	0.4726	0.0838	Improved
1126	Case	0.5140	0.9823	Improved	1026	Case	0.5352	0.8606	Improved
1128	Control	0.4987	0.0508	Improved	1086	Control	0.4549	0.1960	Improved
1365	Control	0.4525	0.3326	Improved	1495	Case	0.5015	0.7915	Improved
1482	Case	0.5277	0.6845	Improved	1597	Case	0.5398	0.8696	Improved
1655	Control	0.4545	0.0392	Improved					

(A) Results of using ${\texttt {NBC}_{\texttt {634}}^{\texttt {(2)}}}$ to remedy ${\texttt {NBC}_{\texttt {634}}^{\texttt {(1)}}}$. (B) Results of using ${\texttt {NBC}_{\texttt {634}}^{\texttt {(1)}}}$ to remedy ${\texttt {NBC}_{\texttt {634}}^{\texttt {(2)}}}$. The 3rd and 3th columns are posterior probabilities of diagnosing instances as “positive” using the main model (i.e., ${\texttt {NBC}_{\texttt {634}}^{\texttt {(1)}}}$ for (A) and ${\texttt {NBC}_{\texttt {634}}^{\texttt {(2)}}}$ for (B)) and the remedying model (i.e., ${\texttt {NBC}_{\texttt {634}}^{\texttt {(2)}}}$ for (A) and ${\texttt {NBC}_{\texttt {634}}^{\texttt {(1)}}}$ for (B)). Only an instance with posterior probability of being diagnosed as “positive” equaling from 0.45 to 0.55 is considered by remedying procedures. Taking the 29th instance (case) for example, ${\texttt {NBC}_{\texttt {634}}^{\texttt {(2)}}}$ accepts “negative” because the posterior probability of diagnosing it as “positive” equals 0.4896 $(<0.5)$; ${\texttt {NBC}_{\texttt {634}}^{\texttt {(1)}}}$ corrects the diagnosis with posterior probability of making correct diagnosis, 0.6215 $(>0.5)$. In this situation, we label the conclusion as *corrected*. For the 1655th instance (control), ${\texttt {NBC}_{\texttt {634}}^{\texttt {(2)}}}$ remedies ${\texttt {NBC}_{\texttt {634}}^{\texttt {(1)}}}$ by improving the posterior probability of making correct diagnosis from 0.5455 $(=1-0.4545>0.5)$ to 0.9608 $(=1-0.0392>0.5455)$. In this situation, the conclusion is labeled as *improved*. Other results can be explained similarly.

### Remedying procedures

As seen, for each dataset, its diagnostic models, ${\texttt {NBC}_{\texttt {study}}^{\texttt {(1)}}}$ and ${\texttt {NBC}_{\texttt {study}}^{\texttt {(2)}}}$, can be regarded as two artificial experts holding different empirical information about the data, and thus can be combined with each other to make remedies. Two remedying procedures are employed as follows: (1) use ${\texttt {NBC}_{\texttt {study}}^{\texttt {(2)}}}$ to remedy ${\texttt {NBC}_{\texttt {study}}^{\texttt {(1)}}}$; (2) use ${\texttt {NBC}_{\texttt {study}}^{\texttt {(1)}}}$ to remedy ${\texttt {NBC}_{\texttt {study}}^{\texttt {(2)}}}$. To avoid over-remedying, only an instance (case or control) with posterior probability of being diagnosed as “positive” larger than 0.45 but less than 0.55 is taken into consideration. Table 1 and Table S2 list all such instances of the five dbGaP studies and corresponding posterior probabilities of being diagnosed as “positive”. By the results, remedying procedures not only correct most of the incorrect diagnoses made by either ${\texttt {NBC}_{\texttt {study}}^{\texttt {(1)}}}$ or ${\texttt {NBC}_{\texttt {study}}^{\texttt {(2)}}}$, but also improve reliability of those correct but doubtful diagnoses by increasing their posterior probabilities of being diagnosed correctly, except the 189th instance of phs000306-JL (Table S2I), for which ${\texttt {NBC}_{\texttt {306}\hbox {-}{} \texttt {JL}}^{\texttt {(1)}}}$ and ${\texttt {NBC}_{\texttt {306}\hbox {-}{} \texttt {JL}}^{\texttt {(2)}}}$ take almost the same posterior probability of making a correct diagnosis.

Finally, Table 2 lists all the 17 incorrect diagnoses (with respect to all NBCs and all datasets) and their posterior probabilities of being diagnosed as “positive” by main models (3rd column of Table 2) and remedying models (4th column). It is seen that all incorrect diagnoses can be desirably corrected. In this sense, remedying procedures can render ${\texttt {NBC}_{\texttt {study}}^{\texttt {(1)}}}$ and ${\texttt {NBC}_{\texttt {study}}^{\texttt {(2)}}}$ to complement mutually and get accuracy up to 100% eventually.

**Table 2:** Performance of remedying procedures on the 17 incorrect diagnoses.

**A**	Instance no.	True status	${\texttt {NBC}_{\texttt {634}}^{\texttt {(1)}}}$	${\texttt {NBC}_{\texttt {634}}^{\texttt {(2)}}}$	Conclusion	**B**	Instance no.	True status	${\texttt {NBC}_{\texttt {634}}^{\texttt {(2)}}}$	${\texttt {NBC}_{\texttt {634}}^{\texttt {(1)}}}$	Conclusion
	No error						29	Case	0.4896	0.6215	Corrected
**C**	Instance no.	True status	${\texttt {NBC}_{\texttt {753}}^{\texttt {(1)}}}$	${\texttt {NBC}_{\texttt {753}}^{\texttt {(2)}}}$	Conclusion	**D**	Instance no.	True status	${\texttt {NBC}_{\texttt {753}}^{\texttt {(2)}}}$	${\texttt {NBC}_{\texttt {753}}^{\texttt {(1)}}}$	Conclusion
	318	Control	0.5171	0.1060	Corrected		414	Control	0.5379	0.1386	Corrected
	1291	Case	0.4465	0.6449	Corrected						
**E**	Instance no.	True status	${\texttt {NBC}_{\texttt {147}}^{\texttt {(1)}}}$	${\texttt {NBC}_{\texttt {147}}^{\texttt {(2)}}}$	Conclusion	**F**	Instance no.	True status	${\texttt {NBC}_{\texttt {147}}^{\texttt {(2)}}}$	${\texttt {NBC}_{\texttt {147}}^{\texttt {(1)}}}$	Conclusion
	1444	Case	0.4680	0.8689	Corrected		1356	Control	0.5486	0.0765	Corrected
	1724	Control	0.6190	0.0276	Corrected						
	1982	Case	0.4549	0.7723	Corrected						
	2153	Case	0.4633	0.9114	Corrected						
**G**	Instance no.	True status	${\texttt {NBC}_{\texttt {517}}^{\texttt {(1)}}}$	${\texttt {NBC}_{\texttt {517}}^{\texttt {(2)}}}$	Conclusion	**H**	Instance no.	True status	${\texttt {NBC}_{\texttt {517}}^{\texttt {(2)}}}$	${\texttt {NBC}_{\texttt {517}}^{\texttt {(1)}}}$	Conclusion
	1354	Case	0.3288	0.5652	Corrected		581	Case	0.4947	0.8736	Corrected
**I**	Instance no.	True status	${\texttt {NBC}_{\texttt {306}\hbox {-}{} \texttt {JL}}^{\texttt {(1)}}}$	${\texttt {NBC}_{\texttt {306}\hbox {-}{} \texttt {JL}}^{\texttt {(2)}}}$	Conclusion	**J**	Instance no.	True status	${\texttt {NBC}_{\texttt {306}\hbox {-}{} \texttt {JL}}^{\texttt {(2)}}}$	${\texttt {NBC}_{\texttt {306}\hbox {-}{} \texttt {JL}}^{\texttt {(1)}}}$	Conclusion
	1114	Case	0.4706	0.9645	Corrected		109	Control	0.5658	0.1494	Corrected
**K**	Instance no.	True status	${\texttt {NBC}_{\texttt {306}\hbox {-}{} \texttt {AA}}^{\texttt {(1)}}}$	${\texttt {NBC}_{\texttt {306}\hbox {-}{} \texttt {AA}}^{\texttt {(2)}}}$	Conclusion	**L**	Instance no.	True status	${\texttt {NBC}_{\texttt {306}\hbox {-}{} \texttt {AA}}^{\texttt {(2)}}}$	${\texttt {NBC}_{\texttt {306}\hbox {-}{} \texttt {AA}}^{\texttt {(1)}}}$	Conclusion
	1006	Control	0.5111	0.1906	Corrected		1107	Case	0.4596	0.7027	Corrected
	1079	Case	0.3978	0.9797	Corrected		2141	Case	0.4224	0.9866	Corrected

(A) Use ${\texttt {NBC}_{\texttt {634}}^{\texttt {(2)}}}$ to remedy ${\texttt {NBC}_{\texttt {634}}^{\texttt {(1)}}}$. (B) Use ${\texttt {NBC}_{\texttt {634}}^{\texttt {(1)}}}$ to remedy ${\texttt {NBC}_{\texttt {634}}^{\texttt {(2)}}}$. (C) Use ${\texttt {NBC}_{\texttt {753}}^{\texttt {(2)}}}$ to remedy ${\texttt {NBC}_{\texttt {753}}^{\texttt {(1)}}}$. (D) Use ${\texttt {NBC}_{\texttt {753}}^{\texttt {(1)}}}$ to remedy ${\texttt {NBC}_{\texttt {753}}^{\texttt {(2)}}}$. (E) Use ${\texttt {NBC}_{\texttt {147}}^{\texttt {(2)}}}$ to remedy ${\texttt {NBC}_{\texttt {147}}^{\texttt {(1)}}}$. (F) Use ${\texttt {NBC}_{\texttt {147}}^{\texttt {(1)}}}$ to remedy ${\texttt {NBC}_{\texttt {147}}^{\texttt {(2)}}}$. (G) Use ${\texttt {NBC}_{\texttt {517}}^{\texttt {(2)}}}$ to remedy ${\texttt {NBC}_{\texttt {517}}^{\texttt {(1)}}}$. (H) Use ${\texttt {NBC}_{\texttt {517}}^{\texttt {(1)}}}$ to remedy ${\texttt {NBC}_{\texttt {517}}^{\texttt {(2)}}}$. (I) Use ${\texttt {NBC}_{\texttt {306}\hbox {-}{} \texttt {JL}}^{\texttt {(2)}}}$ to remedy ${\texttt {NBC}_{\texttt {306}\hbox {-}{} \texttt {JL}}^{\texttt {(1)}}}$. (J) Use ${\texttt {NBC}_{\texttt {306}\hbox {-}{} \texttt {JL}}^{\texttt {(1)}}}$ to remedy ${\texttt {NBC}_{\texttt {306}\hbox {-}{} \texttt {JL}}^{\texttt {(2)}}}$. (K) Use ${\texttt {NBC}_{\texttt {306}\hbox {-}{} \texttt {AA}}^{\texttt {(2)}}}$ to remedy ${\texttt {NBC}_{\texttt {306}\hbox {-}{} \texttt {AA}}^{\texttt {(1)}}}$. (L) Use ${\texttt {NBC}_{\texttt {306}\hbox {-}{} \texttt {AA}}^{\texttt {(1)}}}$ to remedy ${\texttt {NBC}_{\texttt {306}\hbox {-}{} \texttt {AA}}^{\texttt {(2)}}}$.

Sufficient and efficient exploration of rules hidden in available sequencing data is a challenge but also a key to prevention, diagnosis and treatment of complex diseases such as the three top cancers. As expected, it may be increasingly becoming urgent to find SNPs that can be used to make precise diagnoses, rather than only identifying some related or high-risk SNPs^4,23,24^ and then build corresponding models. Our results show this possibility, indicating that moving beyond those initial steps of GWA studies^4^ may be no longer a hard work in the near future!

## Discussion

### Collection of sufficient information about cancers by **Snp2Bin**

The use of Snp2Bin is a key procedure to the performance of making classifications. Without using Snp2Bin, IterMMPC cannot get a good subset of variables, and also OptNBC cannot select proper features to make precise classifications. Taking phs000634 for example, if using Snp2Bin to transform SNPs into 2-value variables, IterMMPC can get a 3274-variable subset, and then OptNBC selects a 268-feature NBC, namely ${\texttt {NBC}_{\texttt {634}}^{\texttt {(1)}}}$, which gets classification accuracy 99.91% according to leave-one-out; In comparison, if not using Snp2Bin, then IterMMPC will get a subset only containing 60 variables, and then OptNBC obtains a 59-feature NBC with accuracy 74.93% only.

### Exclusion of redundant variables for high dimensional SNP genotypes by IterMMPC

For a target variable in a Bayesian network^25^, the parents, children, and spouses are its theoretically optimal features^26^. As a special Bayesian network, NBC needs only the target’s children, which can be identified by the MMPC algorithm. An important working mechanism of MMPC is to use (conditional) independence tests to exclude redundant variables.

Numerically, for each of the six datasets, we check every SNP’s association with cancer risks by computing its (0-order) *p* value used for testing the statistical hypothesis “the SNP is *independent* of cancer risks”. As seen from Fig. 2 and Figs. S3, S4, S5A and S5B, there are many SNPs for which a very high association with cancer risks may not mean a large probability that the corresponding SNP can be selected as a feature, implying such a high association may only be a superficially (not truly) high association.

Such many superficially high associations make it hard to determine an optimal subset of SNPs used for prediction. However, these superficially high associations can be filtered to a great degree by conditioning one or more truly high associated SNPs, as MMPC does. To explain why this works so well, we take phs000517 as an illustration by computing the 1-order *p* value for every SNP when testing “the SNP is *conditionally independent* of cancer risks conditioned on any *one* of those SNPs (except itself) used by ${\texttt {NBC}_{\texttt {517}}^{\texttt {(1)}}}$ or ${\texttt {NBC}_{\texttt {517}}^{\texttt {(2)}}}$”.

By the results (Figs. S5C and S5D), many of the superficially high associations are identified immediately. Hence, we expect that, when 2-order *p* values are used, MMPC can exclude many more redundant variables.

On the other hand, MMPC has an exponential complexity, so it cannot be used directly to select features for a dataset of high dimension (especially when the dimension is larger than one million). Instead, IterMMPC divides all variables into many parts and implements MMPC for every part to update the subset of variables, and then iterates the process until no change occurs. In short words, IterMMPC not only saves computing time, but also finds a small superset of all useful SNPs.

### Selection of optimal features for naive Bayes by **OptNBC**

Our OptNBC algorithm enhances naive Bayes by using a similar idea of constructing the *selective Bayesian clasifier*^27^. If the features are properly used, the resulting classifier will possess robust power of making classifications^19^. Considering the high dimensionality of each dataset, we use the 10-fold cross-validation score (substituting for *leave-one-out* score) to speed up computations. It can be seen from Fig. 4 that the features (selected by using the 10-fold cross-validation score) can make the accuracy (evaluated in the sense of leave-one-out) ascend with only slight fluctuations. This indicates there is no over-fitting in NBCs once the features are properly selected (Supplementary Materials S5). In addition, we use OptNBC also because naive Bayes is simple and has more intuitional probabilistic meanings.

### Number of selected features: from quantity to quality

For a complex disease such as one of these three top cancers, there are no leading SNPs, and per SNP only carries a small amount of information about cancer risks. In some situations, such information also may be swamped by some unknown random factors, and in this case the corresponding SNP will give an opposite effect on predicting cancer risks, needing more other SNPs to offset this opposite effect.

On the other hand, as Matt Ridley said in summarizing the genetic annealing model of Carl Woese: “the organism was not yet an enduring entity, and the genes that ended up in all of us may have come from lots of ‘species’ of creature”^28,29^, we believe that evolution is indeed urging humans (and other species) to mitigate the risk of getting a serious disease by dispersing it to many loci of the micro world, so a large number of SNPs associated with a complex disease have to be identified and used in a better method.

Our results also confirm an early guess of Dr. Gail that *about 300* (=7+10+280) SNPs are needed to dramatically improve risk forecasts for breast cancer^30,31^. The guess of Dr. Gail, however, does not mean we can improve risk forecasts substantially by simply taking $\sim$ 300 (and even more) SNPs that have the highest associations. For example, if using such 300 SNPs, phs000753 can only get accuracy 55.85%, nearly equivalent to guessing cancer risks by tossing coins. Instead, these SNPs should be appropriately chosen from the huge number of SNPs via suitable methods, like our IterMMPC and OptNBC algorithms.

### More information decoded from SNPs

As the third generation of genetic markers, SNP genotypes are expected to contain all information about what one wants to know, such as skin color, gender, ethnicity, temperament, and even sexual orientation, if data on all SNP genotypes are collected properly. For example, to see the gender information hidden in the intersected 170,571 SNPs of phs000634 and phs000753, we regard the 1998/2290 gender labels in this two dbGaP studies as the target data, and then perform Snp2Bin/IterMMPC/OptNBC to make classifications. For phs000634, we get a 385-variable subset and then a 304-feature NBC, which performs “predictions” for gender with accuracy 89.64% according to 10-fold cross-validation; for phs000753, we get a 507-variable subset and then a 311-feature NBC, performing “predictions” with accuracy 92.23%. If all SNPs are pre-collected at the data-gathering phase, the accuracy will be higher. In this sense, those phenotypical information (such as gender) useful for characterizing cancer risks are contained in some SNPs genotypes. This explains why our method can make precise classifications by using SNPs only.

### Application to more complex diseases

Besides the three top cancers, our method can also be applied to many other complex diseases, if corresponding datasets are available. On the one hand, Snp2Bin plays an important role in extracting as much useful information as possible and in making the most efficient use of IterMMPC. On the other hand, among so many SNPs, there is no any leading SNP; in this case, any potential opposite effect of a SNP on making predictions caused by random factors may be remedied by some other SNPs.

### Data availability

All datasets are available through the dbGaP. The main code used in this report is available on https://github.com/lxq2018/dbGaP.

### Data preprocessing

All datasets only consist of the part with restriction of GRU (general research use). For a SNP, its missing values are regarded as chaos states of genotypes. Denote them by an imaginary genotype, instead of simply deleting them or replacing them with imputed data, because such states may stand for certain potential unknowns to be unexplored rather than consequences of some other factors such as precision of sequencers.

### The 2-value coding scheme: **Snp2Bin** algorithm

As Fig. 1A illustrates, Snp2Bin first examines all genotypes (including the imaginary genotype) for a SNP; Then, it transforms the SNP into a 2-value variable by taking 1 for some alleles and 0 for all others; After that, the $\chi ^2$-statistic^32^ of the corresponding contingency table is computed (as its score). Among all such possible coded 2-values variables, the one with the highest score is as the optimal 2-value variable for this SNP. This scheme borrows in part the idea of transforming a multi-class attribute into a binary variable^33^ and can increase the power of $\chi ^2$-tests involved in subsequent process of building models, so it is a key to implement IterMMPC and OptNBC/SubOptNBC. This is because, for a SNP related to the target, one or more of its genotypes may be only weakly dependent on (or even nearly independent of) the cancer, and such genotypes increase the statistical degrees of freedom for the corresponding $\chi ^2$-test, leading further to a false conclusion about the dependence between this SNP and the cancer. Snp2Bin enhances the ability to detect such dependence.

Moreover, it can be verified that, for any SNP independent of the cancer, the corresponding 2-value variable must also be independent of this cancer. In fact, let *T* and *X* be two random variables, taking $\{t_1,\ldots ,t_k\}$ and $\{x_1,\ldots ,x_\ell \}$, respectively. If *T* and *X* are independent, $P(T = t_i, X = x_j) = P(T = t_i) P(X = x_j)$ holds for any $i = 1,\ldots ,k$ and $j = 1,\ldots ,\ell$. Let *Y* be one of the 2-value variables of *X*, defined as taking 1 if $X\in \mathcal {X}_1$ and taking 0 otherwise, where $\mathcal {X}_1$ and $\mathcal {X}_0$ are two (nonempty) exclusive and exhaustive subsets of $\{x_1,\ldots ,x_\ell \}$. Then, for any $t\in \{t_1,\ldots ,t_k\}$ and $y\in \{1,0\}$, we have

$$\begin{aligned} P(T = t, Y = y)&= P\left( T = t,\cup _{x\in \mathcal {X}_y}\{X = x\}\right) =\textstyle \sum \limits _{x\in \mathcal {X}_y} P(T = t,X = x) =\textstyle \sum \limits _{x\in \mathcal {X}_y} P(T = t) P(X = x)\\&= P(T = t) \textstyle \sum \limits _{x\in \mathcal {X}_y}P(X = x) = P(T = t) P\left( \cup _{x\in \mathcal {X}_y}\{X = x\}\right) =P(T=t,Y=y). \end{aligned}$$

It follows that *T* and *Y* are also independent. This indicates (1) unrelated SNPs will never enter our NBC models, and (2) the information that a SNP carries about the cancer will be encoded by the 2-value variable as much as possible.

### Reduction of search space for NBC: IterMMPC algorithm

As a simple Bayesian network^25^, all the features in an NBC are children of the target (status of lung cancer or breast cancer or prostate cancer). Considering the number of SNPs is very huge, up to half a million and even larger, we use IterMMPC  to reduce the search space before looking for the optimal NBC. MMPC^34,35^ is a state-of-the-art algorithm used for finding the parents (direct causes) and children (direct efforts) of the target. Its computational complexity is exponential to the number of parents and children, so we divide the feature set into a number of groups and update each group individually by applying MMPC to it. Iterate this process until no change occurs. Figure 1B describes this divide-and-conquer strategy schematically. To avoid over-excluding useful attributes, the two parameters of MMPC, “threshold” and “maxK”, are taken as 0.1 and 2, respectively.

### Optimal NBC discovery: **OptNBC** algorithm

IterMMPC  gets a superset of attributes of a target. Specifically, this superset contains 3274 attributes for phs000634, 1298 attributes for phs000753, 4128 attributes for phs000147, 1863 attributes for phs000517, 3919 attributes for phs000306-JL, and 24,457 attributes for phs000306-AA. Based on these filtered attributes, OptNBC starts from an empty NBC. As Fig. 1C shows, for each attribute, add it tentatively to the current NBC and then compute the product of posterior probabilities of making correct diagnoses (just as the likelihood function in some sense; or equivalently, its logarithm) as its score. Add the attribute with the highest score to the current NBC to update the forward phase of OptNBC until the score no longer increases. Then, remove any attribute tentatively from the current NBC and then compute its score, deleting the attribute with the lowest score to update the backward phase until the score begins to decrease.

### Alternative to **OptNBC**: **SubOptNBC** algorithm

SubOptNBC  is an alternative algorithm to OptNBC in searching a good NBC. It simply replaces OptNBC by adding the attribute with the second highest score to the NBC in the forward phase. The NBCs searched by OptNBC and SubOptNBC can be regarded as two different experts of making diagnoses with different empirical information in a sense.

## Supplementary information

Supplementary Information.
