# Supplementary material for: Precise diagnosis of three top cancers using dbGaP data
Source: Sci Rep. 2021 Jan 12;11:823. doi: 10.1038/s41598-020-80832-x (PMC7804208; doi:10.1038/s41598-020-80832-x)
Supplement: Supplementary file 1 — Supplementary Information. [file 41598_2020_80832_MOESM1_ESM.pdf]

Supplementary Materials for

Precise diagnosis of three top cancers using dbGaP data

Xu-Qing Liu, Xin-Sheng Liu, Jian-Ying Rong, Feng Gao, Yan-Dong Wu, Chun-Hua Deng, Hong-Yan Jiang, Xiao-Feng Li, Ye-Qin Chen, Zhi-Guo Zhao, Yu-Ting Liu, Hai-Wen Chen, Jun-Liang Li, Yu Huang, Cheng-Yao Ji, Wen-Wen Liu, Xiao-Hu Luo, Li-Li Xiao

This PDF file includes:

Materials and Methods

Algorithms S1 to S3

Figures S1 to S5

Tables S1 to S7

Materials and Methods

S1 Naive Bayes classifier

As a simple Bayesian network[24], the naive Bayes classifier (NBC)[18] has the following graphical structure:

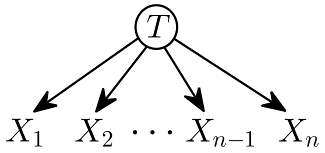

Given the target T, its features $X_1, X_2 \cdots, X_n$ are conditionally independent. Although this local independence assumption is often violated in practice, NBC still performs "unreasonably" robust[19]. In addition, Section S5 will explain why no overfitting problem occurs in NBCs with proper features. Therefore, we employ NBC to explore and build precise diagnostic modles.

Anyway, NBC can play its advantages in making classifications only when the associated features are properly used. As seen, for every dataset, the number of potential attributes is very huge, up to half a million or even larger, so it is necessary to reduce the search space appropriately before starting to select features for NBC. To do this, it is important to use a suitable coding scheme for SNPs.

S2 The 2-value coding scheme: Snp2Bin algorithm

After making preliminary attempts, we find the association between almost every 3- or "4-genotype" SNP and the target (status of lung cancer or breast cancer or prostate cancer) is unexpectedly low, although many SNPs are of statistical significance. In general, a SNP has three genotypes. However, some genotypes with very low proportion may not appear in a dataset, leading to some 1- or 2-genotype SNPs. In addition, there are many missing values (about 10% and even more of the total sequencing results) for SNPs in the six datasets. We regard them as a chaos or mixed state of genotypes, instead of deleting them simply or replacing them with imputed data. Such a state is then treated as an imaginary genotype, which stands for potential unknowns to be unexplored, rather than as a consequence of some other factors like precision of sequencers.

The reason for this is that, for a SNP related to the target, one or more of its genotypes may be only weakly dependent on (or even nearly independent of) the cancer, and such genotypes increase the statistical degrees of freedom for the corresponding χ^2-test, leading further to a false conclusion about the dependence between this SNP and the cancer.

To solve this problem, we employ in part the idea of transforming a multi-class attribute into a 2-value variable[32] to increase power of χ^2-tests. Specifically, for a SNP, let X be a 2-value variable taking 1 for some genotypes and 0 for all others and, among all such 2-value variables, select the one having the maximal χ^2-statistic[31] with respect to the cancer. In fact, our algorithm needs to test many hypotheses of the form "T and X are independent conditioned on Y", where Y is the conditioning set containing one or more variables. If X and every variable in Y are 3-value variables, the degree of freedom of the corresponding χ^2- or G^2-statistic will be $(2-1) \times (3-1) \times 3^{|Y|} = 2 \times 3^{|Y|}$; In comparison, if X and every variable in Y are 2-value variables, the degree of freedom will decrease sharply to $(2-1) \times (2-1) \times 2^{|Y|} = 2^{|Y|}$. This means that Snp2Bin is critical in improving the efficiency of our algorithm. For example, if Y contains three variables, the degrees of freedom will be 54 and 8, respectively, for the two cases. Algo. S1 describes the pseudocode of the resulting algorithm,

namely “Snp2Bin”.

By direct analysis, it can be verified that, for any SNP independent of the cancer, the corresponding 2-value variable must also be independent of this cancer. It follows that T and Y are also independent. This indicates (i) unrelated SNPs will never enter our NBC models, and (ii) the information that a SNP carries about the cancer will be encoded by the corresponding 2-value variable as much as possible.

As an illustration, we take the SNP, rs7524868 of phs000634, as an example (Fig. 1A). As seen, the 2-value coding scheme combines the genotypes such that the information about the cancer can be integrated in a better way, and hence improves the association of the coded variables in most situations.

Algo. S1. Snp2Bin **algorithm:** transforming genotypes into 2-value variables in the sense of getting the largest mutual information or χ^2-statistic, denoted by the symbol "I" in Line 11.

Procedure: $[\boldsymbol{b}, \boldsymbol{c}] \leftarrow \mathsf{Snp2Bin}(\boldsymbol{a}, \boldsymbol{s})$

Input: $\boldsymbol{a} \triangleq (a_1, \cdots, a_n)^T$ is a vector of genotypes, while $\boldsymbol{s} \triangleq (s_1, \cdots, s_n)^T$ is a case-control status vector, in which a_i and s_i are from the i-th instance.

Output: $\boldsymbol{b}$ is a 2-value vector of coding $\boldsymbol{a}$; $\boldsymbol{c}$ is a cell of indicating how genotypes are coded.

1. $\boldsymbol{\gamma} \leftarrow$ unique of $\boldsymbol{a}$
2. **foreach** nonempty set $\boldsymbol{\gamma}_j \subsetneqq \boldsymbol{\gamma}$ **do**
3.   **for** i taking from 1 to n **do**
4.   **if** $a_i \in \boldsymbol{\gamma}_j$ **then**
5. $\beta_i \leftarrow 1$
6.   **else**
7. $\beta_i \leftarrow 0$
8.   **end**
9.   **end**
10. $\boldsymbol{\beta}_j \leftarrow (\beta_1, \cdots, \beta_n)^T$
11. $\alpha_j \leftarrow I(\boldsymbol{\beta}_j, \boldsymbol{s})$
12. **end**
13. $\ell \leftarrow \arg\max_j\{\alpha_j\}$
14. **return** $\boldsymbol{b} \leftarrow \boldsymbol{\beta}_\ell$ and $\boldsymbol{c} \leftarrow \{\boldsymbol{\gamma}_\ell, \boldsymbol{\gamma} \backslash \boldsymbol{\gamma}_\ell\}$

S3 Reduction of search space for NBC: IterMMPC algorithm

To reduce the search space of NBC, we choose to use the MMPC (max-min parents and children) algorithm[33,34]. Here, we briefly introduce why we choose MMPC to make search space reduction. Let us first see a simple Bayesian network as follows ($X_1, \cdots, X_8$ are random variables or called *nodes*):

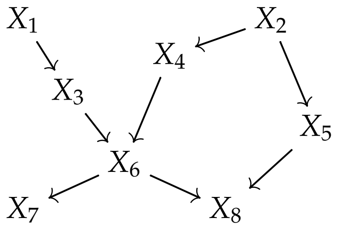

For X_4, (i) in *graphical* sense, X_2 is its parent, X_6 is its child, and X_3 is its spouse, they block all information channels from X_4 to other nodes; (ii) in *probabilistic* sense, X_4 is conditionally independent of all other variables given $\{X_2, X_6, X_3\} \triangleq \boldsymbol{M}$. In theory of Bayesian networks, $\boldsymbol{M}$ is called a Markov blanket[24] or, under the faithfulness condition, the Markov boundary of X_4. Pellet and Elisseeff[25] proved that $\boldsymbol{M}$ (the set of parents, children and spouses) is the theoretically optimal set of features of X_4. NBC needs only children of the target, so we use the MMPC algorithm here.

Considering that the computational complexity of MMPC is linear to the number of all variables but exponential to the number of parents and children, we apply a divide-and-conquer strategy by dividing all SNP attributes randomly into a number of groups and implementing MMPC over each group to filter redundant variables. Iterate this procedure until no change occurs. The resulting algorithm, namely "IterMMPC", is described in Algo. S2. To avoid filtering useful SNPs out, we take the two parameters, `threshold` and `maxK`, of MMPC as 0.1 and 2, respectively. This algorithm is expected to obtain a superset of the features for our NBC models.

Algo. S2. IterMMPC **algorithm:** iteratively using the MMPC algorithm to select features of the target.

Procedure: $[\mathcal{F}, \boldsymbol{B}] \leftarrow \text{IterMMPC}(\boldsymbol{B}, \boldsymbol{s}, k)$

Input: $\boldsymbol{B}$ is the data matrix, with each column being produced by Algo. S1; $\boldsymbol{s}$ is the same as defined in Algo. S1; k is the maximal number of variables in per partition, taken as 10 by default.

Output: $\mathcal{F}$ is a superset of causal nodes for the target; $\boldsymbol{B}$ is updated data matrix corresponding to $\mathcal{F}$.

$\mathcal{F} \leftarrow$ attribute set of $\boldsymbol{B}$
while 1 **do**
    divide $\mathcal{F}$ into $\lceil |\mathcal{F}|/k \rceil \triangleq q$ groups, $\mathcal{F}_1, \cdots, \mathcal{F}_q$, such that each contains at most k attributes
    foreach group $\mathcal{F}_j$ **do**
        $\mathcal{F}_j \leftarrow$ output of MMPC over $\mathcal{F}_j$ with respect to $\boldsymbol{B}$ and $\boldsymbol{s}$
    end
    $\mathcal{F} \leftarrow \cup_{j=1}^{q} \mathcal{F}_j$
    $\boldsymbol{B} \leftarrow$ updated data matrix corresponding to $\mathcal{F}$
    if $\mathcal{F}$ remains unchanged **then**
        break
    end
end
return $\mathcal{F}$ and $\boldsymbol{B}$

S4 NBC discovery: OptNBC and SubOptNBC algorithms

After applying IterMMPC, a further feature selection procedure is still required. Now, we first use a score-based method to build our NBCs, namely OptNBC, for which the pseudocode is presented in Algo. S3. The algorithm consists of two phases: in its *forward* phase, attributes are added to the candidate feature set one by one rendering the fastest increase of scores; in its *backward* phase, the redundant variables are removed one by one. Here, the score of an NBC is defined as the product of the posterior probabilities of making correct diagnoses (or equivalently, its logarithm) according to 10-fold cross-validation.

Theoretically, the output of MMPC should be the optimal set of features for the target. However, MMPC is used in partitioned data iteratively instead of in the whole data directly, so there may be some redundant variables remaining in the output of our IterMMPC algorithm. On the other hand, in an NBC model, some parents will not be used as features, leading to a potential compensation from some children or other variables. OptNBC aims to do this in a simple but efficient way.

SubOptNBC is an alternative algorithm to OptNBC in searching a good NBC. We build this algorithm because we want to explore the information hidden in data more sufficiently. SubOptNBC simply replaces OptNBC by adding the attribute with the second highest score to the NBC in each step of the forward phase, so its pseudocode is omitted here. The NBCs searched by OptNBC and SubOptNBC can be viewed as two different experts of making diagnoses with different empirical information.

Algo. S3. OptNBC **algorithm:** searching optimal NBC. It consists of two phases: Lines 1~12 describe the forward phase; Lines 13~23 are for the backward phase. In Line 6 and Line 17, $\mathcal{J}_{\text{FW}}$ and $\mathcal{J}_{\text{BW}}$ are defined as $\mathcal{J}_{\text{FW}} \triangleq \{j : f_j \in \mathcal{F} \setminus \mathcal{G}\}$ and $\mathcal{J}_{\text{BW}} \triangleq \{j : g_j \in \mathcal{G}\}$, respectively. Replacing Line 6 by $\ell \leftarrow \arg\max_{j \in \mathcal{J}_{\text{FW}} \setminus \{\arg\max_{j \in \mathcal{J}_{\text{FW}}} \{\alpha_j\}\}} \{\alpha_j\}$ before ending the forward phase, the resulting algorithm is called SubOptNBC.

Procedure: $\mathcal{M} \leftarrow$ OptNBC($\mathcal{F}, \boldsymbol{B}, \boldsymbol{s}$)

Input: $\mathcal{F}$ and $\boldsymbol{B}$ are outputs of Algorithm S2; $\boldsymbol{s}$ is the same as defined in Algorithm S1.

Output: $\mathcal{M}$ is the searched optimal NBC, in which only the graphical structure is used when performing leave-one-out or 10-fold cross-validation.

1. $\mathcal{G} \leftarrow \varnothing$ and $\alpha \leftarrow 0$
2. **while** 1 **do**
3.   **foreach** attribute $f_j \in \mathcal{F} \setminus \mathcal{G}$ **do**
4. $\alpha_j \leftarrow$ score of NBC over $\mathcal{G} \cup \{f_j\}$
5.   **end**
6. $\ell \leftarrow \arg\max_{j \in \mathcal{J}_{\text{FW}}} \{\alpha_j\}$
7.   **if** $\alpha_\ell > \alpha$ **then**
8. $\mathcal{G} \leftarrow \mathcal{G} \cup \{f_\ell\}$ and $\alpha \leftarrow \alpha_\ell$
9.   **else**
10.   **break**
11.   **end**
12. **end**
13. **while** 1 **do**
14.   **foreach** attribute $g_j \in \mathcal{G}$ **do**
15. $\alpha_j \leftarrow$ score of NBC over $\mathcal{G} \setminus \{g_j\}$
16.   **end**
17. $\ell \leftarrow \arg\max_{j \in \mathcal{J}_{\text{BW}}} \{\alpha_j\}$
18.   **if** $\alpha_\ell \geqslant \alpha$ **then**
19. $\mathcal{G} \leftarrow \mathcal{G} \setminus \{g_\ell\}$ and $\alpha \leftarrow \alpha_\ell$
20.   **else**
21.   **break**
22.   **end**
23. **end**
24. **return** $\mathcal{M} \leftarrow$ NBC with $\mathcal{G}$ as its features

S5 An explanation about why no over-fitting in NBCs with proper features

Taking the following model as an example:

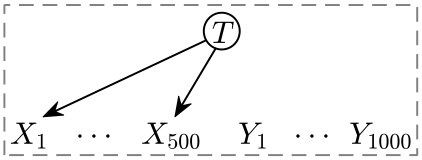

in which T is the target (class) variable, $X_1, \cdots, X_{500}$ are the features of T, and $Y_1, \cdots, Y_{1000}$ are redundant (independent) variables; all parameters are randomly created. For this model, 1000 data points are randomly generated, based on which a simulation study is made with respect to *fitting*, *leave-one-out* and *10-fold cross-validation* as follows: (i) using m features to classify T for m = 100, 200, $\cdots$, 500; (ii) using n redundant variables to classify T for n = 200, 400, $\cdots$, 1000. The values of accuracy, sensitivity, specification and MCC are listed in Tables S3–S6, respectively. By the results, it concludes that

- When using m true features to make classifications, NBC performs better and better along with the increase of m (upto near 100%-accuracy), and there is almost no difference between fitting and leave-one-out/10-fold cross-validation, showing no over-fitting problem occurs.

- When using n redundant variables to make classifications, under the fitting criterion, serious over-fitting occurs, while under leave-one-out/10-fold cross-validation, predicting the status of T is nearly equivalent to guessing it by tossing a coin. This indicates over-fitting cannot occur under leave-one-out/10-fold cross-validation.

In short words, classifications may be made with accuracy upto or near 100% without over-fitting, as long as the features are correctly pre-determined and the classifier is properly selected.

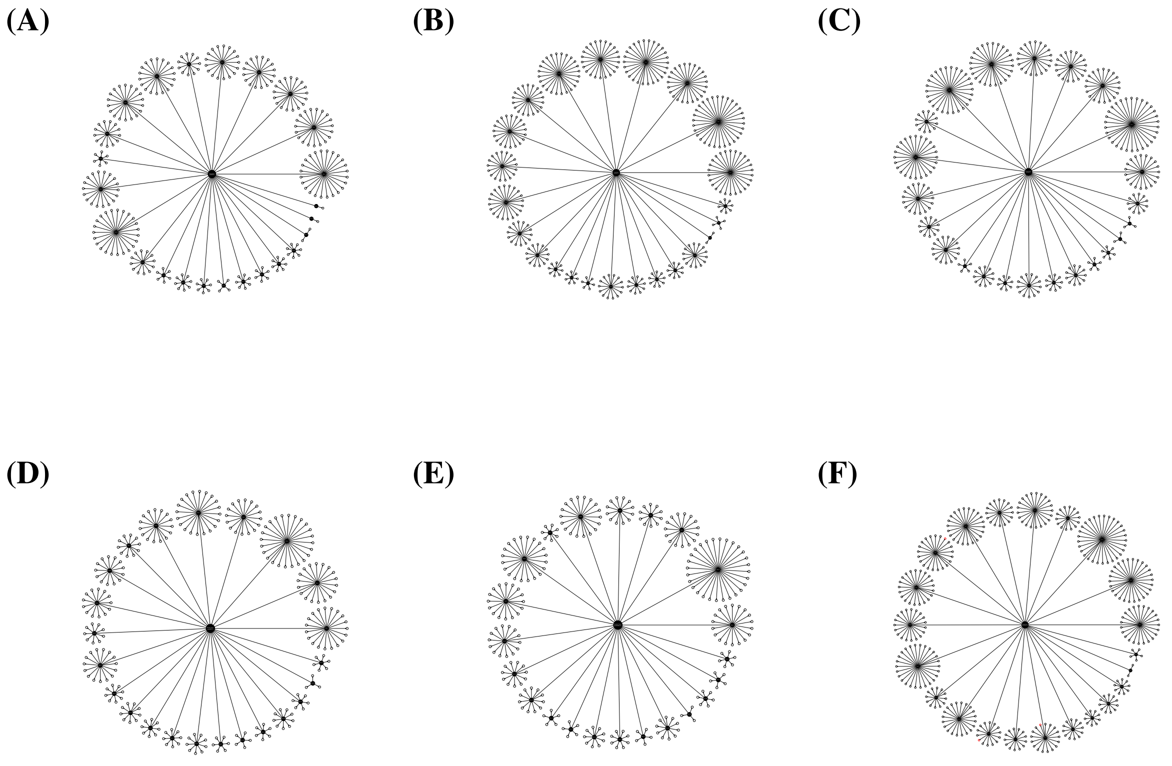

Fig. S1. Diagnostic models based on the OptNBC algorithm (all the SNPs can be seen clearly by enlarging the figure; "Chr" denotes "chromosome")**.** **(A)** Model $NBC_{634}^{(1)}$. It consists of 268 SNPs distributed on all chromosomes, getting accuracy 100% according to leave-one-out. **(B)** Model $NBC_{753}^{(1)}$. It consists of 343 SNPs distributed on all chromosomes except Y, getting accuracy 99.91% according to leave-one-out. **(C)** Model $NBC_{147}^{(1)}$. It consists of 318 SNPs distributed on all chromosomes except Y, getting accuracy 99.83% according to leave-one-out. **(D)** Model $NBC_{517}^{(1)}$. It consists of 255 SNPs distributed on all chromosomes except Y, getting accuracy 99.93% according to leave-one-out. **(E)** Model $NBC_{306\text{-JL}}^{(1)}$. It consists of 242 SNPs distributed on all chromosomes except X and Y, getting accuracy 99.94% according to leave-one-out. **(F)** Model $NBC_{306\text{-AA}}^{(1)}$. It consists of 352 SNPs distributed on all chromosomes except Y, getting accuracy 99.93% according to leave-one-out.

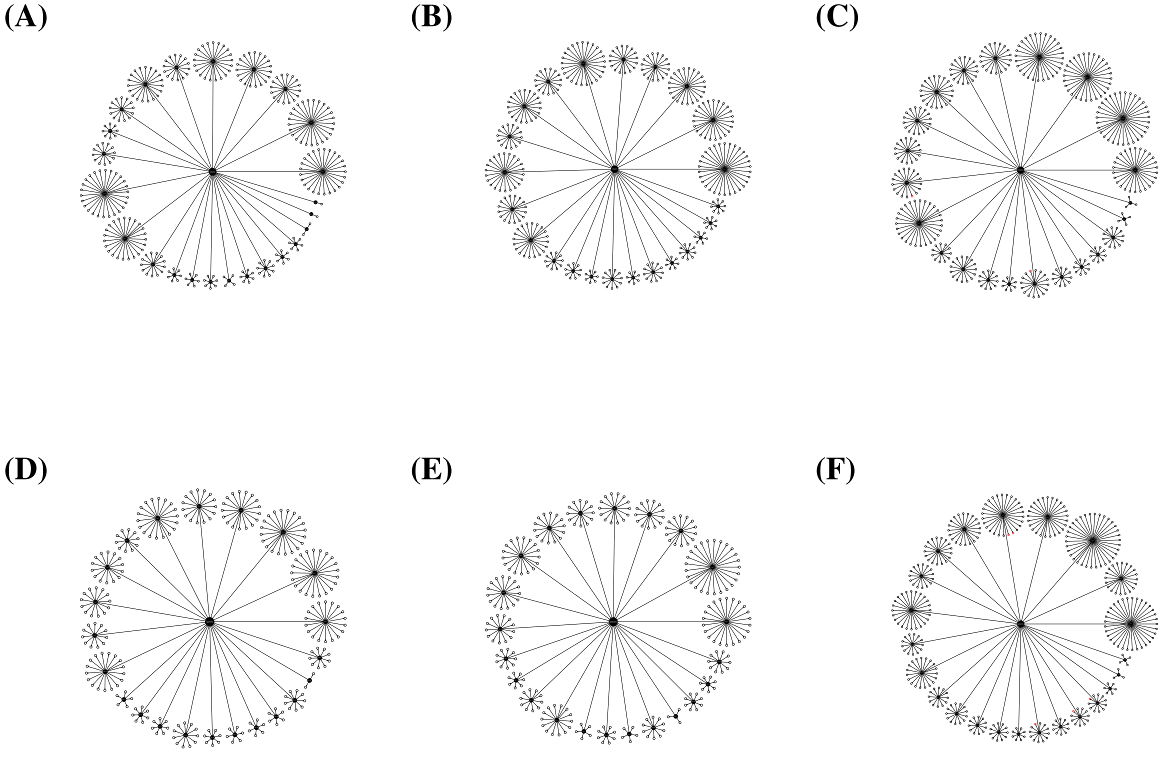

Fig. S2. Diagnostic models based on the SubOptNBC algorithm. **(A)** Model $NBC^{(2)}_{634}$. It consists of 290 SNPs distributed on all chromosomes, getting accuracy 99.95% according to leave-one-out. **(B)** Model $NBC^{(2)}_{753}$. It consists of 329 SNPs distributed on all chromosomes except Y, getting accuracy 99.96% according to leave-one-out. **(C)** Model $NBC^{(2)}_{147}$. It consists of 307 SNPs distributed on all chromosomes except Y, getting accuracy 99.96% according to leave-one-out. **(D)** Model $NBC^{(2)}_{517}$. It consists of 249 SNPs distributed on all chromosomes except 22 and Y, getting accuracy 99.93% according to leave-one-out. **(E)** Model $NBC^{(2)}_{306\text{-JL}}$. It consists of 258 SNPs distributed on all chromosomes except X and Y, getting accuracy 99.94% according to leave-one-out. **(F)** Model $NBC^{(2)}_{306\text{-AA}}$. It consists of 367 SNPs distributed on all chromosomes except Y, getting accuracy 99.93% according to leave-one-out.

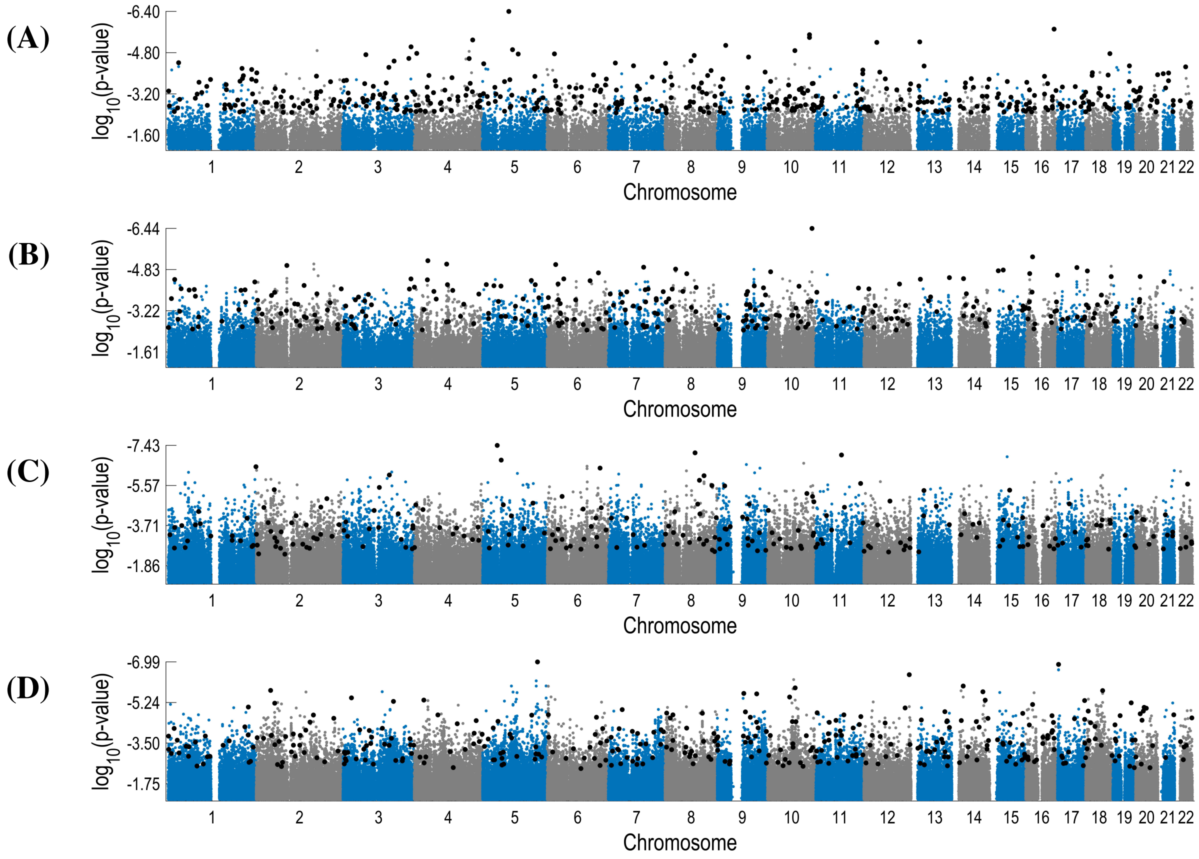

Fig. S3. Log$_{10}$(p-value) of SNPs associated with cancer risks. **(A)** Log$_{10}$(p-value) of all SNPs associated with lung cancer risk based on phs000753 and those used in NBC$^{(1)}_{753}$. **(B)** Log$_{10}$(p-value) of all SNPs associated with breast cancer risk based on phs000147 and those used in NBC$^{(1)}_{147}$. **(C)** Log$_{10}$(p-value) of all SNPs associated with prostate cancer risk based on JL of phs000306 and those used in NBC$^{(1)}_{306\text{-JL}}$. **(D)** Log$_{10}$(p-value) of all SNPs associated with prostate cancer risk based on AA of phs000306 and those used in NBC$^{(1)}_{306\text{-AA}}$.

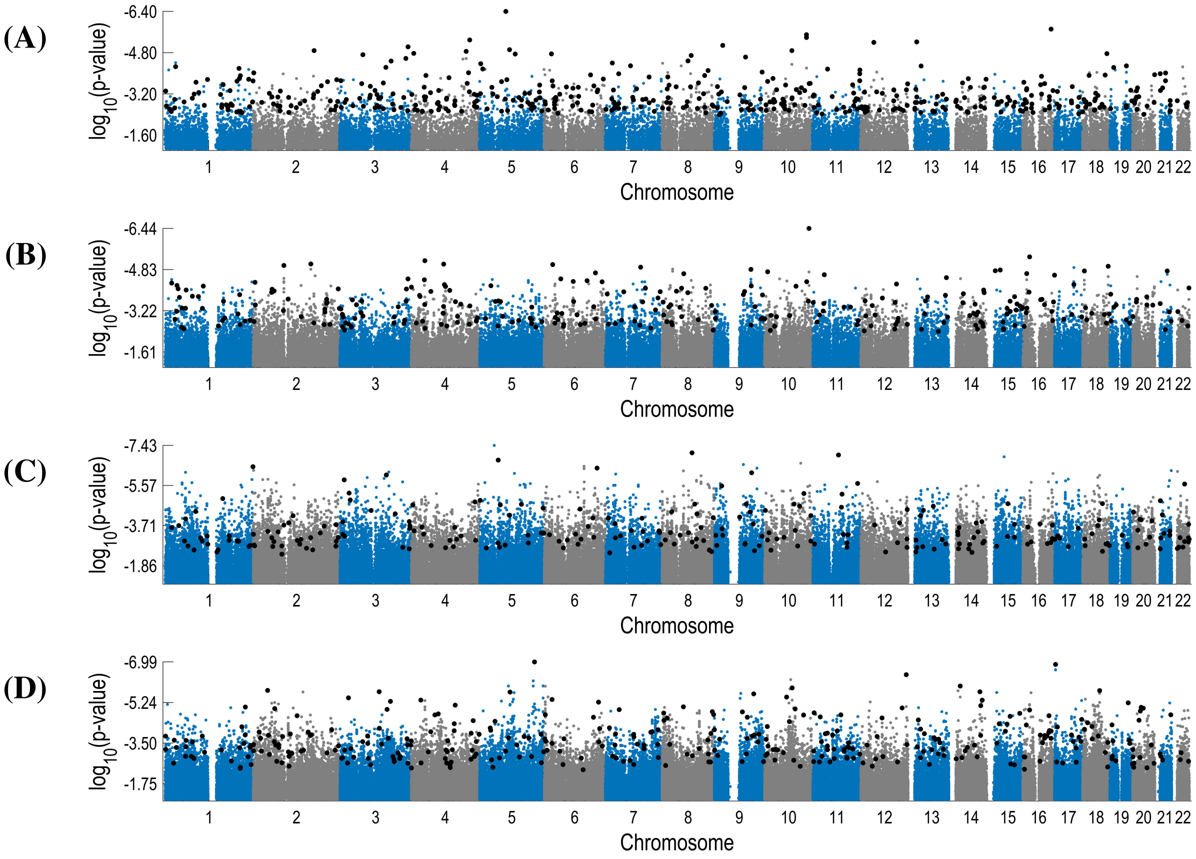

Fig. S4. $\mathbf{Log_{10}}$(*p*-value) of SNPs associated with cancer risks. **(A)** Log_{10}(*p*-value) of all SNPs associated with lung cancer risk based on phs000753 and those used in $\mathrm{NBC}_{753}^{(2)}$. **(B)** Log_{10}(*p*-value) of all SNPs associated with breast cancer risk based on phs000147 and those used in $\mathrm{NBC}_{147}^{(2)}$. **(C)** Log_{10}(*p*-value) of all SNPs associated with prostate cancer risk based on JL of phs000306 and those used in $\mathrm{NBC}_{306\text{-JL}}^{(2)}$. **(D)** Log_{10}(*p*-value) of all SNPs associated with prostate cancer risk based on AA of phs000306 and those used in $\mathrm{NBC}_{306\text{-AA}}^{(2)}$.

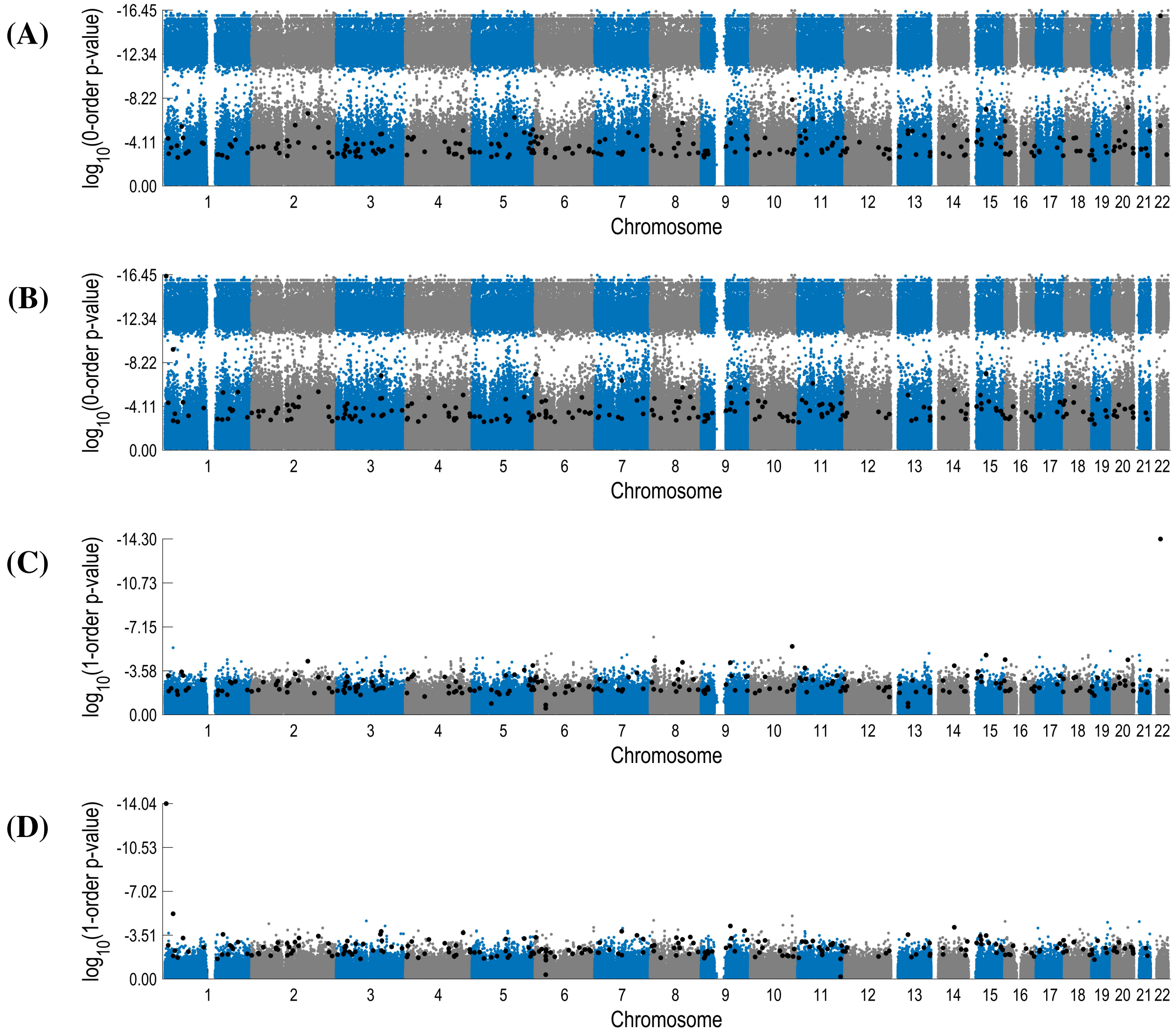

Fig. S5. Log_{10}(*p*-value) of SNPs associated with breast cancer risk based on phs000517. (A) Log_{10}(0-order *p*-value) of all SNPs and those used in $\mathrm{NBC}_{517}^{(1)}$, in which 0-order *p*-values are for testing unconditional independence. **(B)** Log_{10}(0-order *p*-value) of all SNPs and those used in $\mathrm{NBC}_{517}^{(2)}$, in which 0-order *p*-values are for testing unconditional independence. **(C)** Log_{10}(1-order *p*-value) of all SNPs and those used in $\mathrm{NBC}_{517}^{(1)}$, in which 1-order *p*-values are for testing independence conditioned on one of the SNPs in $\mathrm{NBC}_{517}^{(1)}$. **(D)** Log_{10}(1-order *p*-value) of all SNPs and those used in $\mathrm{NBC}_{517}^{(2)}$, in which 1-order *p*-values are for testing independence conditioned on one of the SNPs in $\mathrm{NBC}_{517}^{(2)}$.

Table S1. Classification performance of NBCs according to leave-one-out. **(A)** Confusion matrix of $NBC^{(1)}_{634}$; **(B)** Confusion matrix of $NBC^{(2)}_{634}$; **(C)** Confusion matrix of $NBC^{(1)}_{753}$; **(D)** Confusion matrix of $NBC^{(2)}_{753}$; **(E)** Confusion matrix of $NBC^{(1)}_{147}$; **(F)** Confusion matrix of $NBC^{(2)}_{147}$; **(G)** Confusion matrix of $NBC^{(1)}_{517}$; **(H)** Confusion matrix of $NBC^{(2)}_{517}$; **(I)** Confusion matrix of $NBC^{(1)}_{306\text{-JL}}$; **(J)** Confusion matrix of $NBC^{(2)}_{306\text{-JL}}$; **(K)** Confusion matrix of $NBC^{(1)}_{306\text{-AA}}$; **(L)** Confusion matrix of $NBC^{(2)}_{306\text{-AA}}$.

(A)

$NBC^{(1)}_{634}$		Truth	
		Case	Control
Test	Positive	946	0
	Negative	0	1052

(B)

$NBC^{(2)}_{634}$		Truth	
		Case	Control
Test	Positive	945	0
	Negative	1	1052

(C)

$NBC^{(1)}_{753}$		Truth	
		Case	Control
Test	Positive	1152	1
	Negative	1	1136

(D)

$NBC^{(2)}_{753}$		Truth	
		Case	Control
Test	Positive	1153	1
	Negative	0	1136

(E)

$NBC^{(1)}_{147}$		Truth	
		Case	Control
Test	Positive	1142	1
	Negative	3	1141

(F)

$NBC^{(2)}_{147}$		Truth	
		Case	Control
Test	Positive	1145	1
	Negative	0	1141

(G)

$NBC^{(1)}_{517}$		Truth	
		Case	Control
Test	Positive	698	0
	Negative	1	667

(H)

$NBC^{(2)}_{517}$		Truth	
		Case	Control
Test	Positive	698	0
	Negative	1	667

(I)

$NBC^{(1)}_{306\text{-JL}}$		Truth	
		Case	Control
Test	Positive	828	0
	Negative	1	836

(J)

$NBC^{(2)}_{306\text{-JL}}$		Truth	
		Case	Control
Test	Positive	829	1
	Negative	0	835

(K)

$NBC^{(1)}_{306\text{-AA}}$		Truth	
		Case	Control
Test	Positive	1430	1
	Negative	1	1423

(L)

$NBC^{(2)}_{306\text{-AA}}$		Truth	
		Case	Control
Test	Positive	1429	0
	Negative	2	1424

Table S2. Performance of remedying procedures (Table 1 continued). **(C)** $NBC^{(2)}_{753}$ remedies $NBC^{(2)}_{753}$; **(D)** $NBC^{(1)}_{753}$ remedies $NBC^{(2)}_{753}$; **(E)** $NBC^{(2)}_{147}$ remedies $NBC^{(1)}_{147}$; **(F)** $NBC^{(1)}_{147}$ remedies $NBC^{(2)}_{147}$; **(G)** $NBC^{(2)}_{517}$ remedies $NBC^{(1)}_{517}$; **(H)** $NBC^{(1)}_{517}$ remedies $NBC^{(2)}_{517}$; **(I)** $NBC^{(2)}_{306\text{-JL}}$ remedies $NBC^{(1)}_{306\text{-JL}}$; **(J)** $NBC^{(1)}_{306\text{-JL}}$ remedies $NBC^{(2)}_{306\text{-JL}}$; **(K)** $NBC^{(2)}_{306\text{-AA}}$ remedies $NBC^{(1)}_{306\text{-AA}}$; **(L)** $NBC^{(1)}_{306\text{-AA}}$ remedies $NBC^{(2)}_{306\text{-AA}}$.

(C)

Instance No.		$NBC^{(1)}_{753}$	$NBC^{(2)}_{753}$	Concl.
318	(Ctrl)	0.5171	0.1060	Corrected
575	(Ctrl)	0.4588	0.1223	Improved
778	(Ctrl)	0.4888	0.0608	Improved
800	(Ctrl)	0.4778	0.0160	Improved
1300	(Case)	0.5355	0.6472	Improved
1781	(Case)	0.5388	0.7085	Improved
1918	(Case)	0.5378	0.8241	Improved
2009	(Case)	0.5338	0.7095	Improved
2059	(Case)	0.5118	0.7765	Improved

(D)

Instance No.		$NBC^{(2)}_{753}$	$NBC^{(1)}_{753}$	Concl.
80	(Ctrl)	0.4619	0.0750	Improved
212	(Ctrl)	0.4980	0.1532	Improved
414	(Ctrl)	0.5379	0.1386	Corrected
526	(Ctrl)	0.4695	0.0456	Improved
739	(Ctrl)	0.4834	0.0443	Improved
1102	(Ctrl)	0.4857	0.0612	Improved
1278	(Case)	0.5298	0.7456	Improved
1282	(Case)	0.5140	0.9252	Improved
1327	(Case)	0.5465	0.9260	Improved
1454	(Case)	0.5397	0.9037	Improved
1467	(Case)	0.5167	0.5590	Improved
1988	(Case)	0.5473	0.9084	Improved
1991	(Case)	0.5073	0.6515	Improved
2001	(Case)	0.5378	0.9134	Improved
2079	(Case)	0.5256	0.8393	Improved
2194	(Case)	0.5218	0.9009	Improved

(E)

Instance No.		$NBC^{(1)}_{147}$	$NBC^{(2)}_{147}$	Concl.
419	(Case)	0.5448	0.8086	Improved
1323	(Case)	0.5006	0.8739	Improved
1444	(Case)	0.4680	0.8689	Corrected
1936	(Case)	0.5389	0.7347	Improved
1956	(Ctrl)	0.4631	0.0718	Improved
1982	(Case)	0.4549	0.7723	Corrected
2153	(Case)	0.4633	0.9114	Corrected

(F)

Instance No.		$NBC^{(2)}_{147}$	$NBC^{(1)}_{147}$	Concl.
281	(Ctrl)	0.4564	0.1147	Improved
433	(Case)	0.5436	0.9379	Improved
441	(Case)	0.5149	0.5864	Improved
568	(Ctrl)	0.4590	0.2063	Improved
620	(Ctrl)	0.4844	0.1181	Improved
1046	(Case)	0.5203	0.9746	Improved
1356	(Ctrl)	0.5486	0.0765	Corrected
1521	(Ctrl)	0.4631	0.0793	Improved

(G)

Instance No.		$NBC^{(1)}_{517}$	$NBC^{(2)}_{517}$	Concl.
1038	(Case)	0.5300	0.8261	Improved
1276	(Ctrl)	0.4560	0.3984	Improved

(H)

Instance No.		$NBC^{(2)}_{517}$	$NBC^{(1)}_{517}$	Concl.
46	(Case)	0.5161	0.8553	Improved
370	(Case)	0.5493	0.6435	Improved
383	(Case)	0.5068	0.9606	Improved
581	(Case)	0.4947	0.8736	Corrected

(I)

Instance No.		$NBC^{(1)}_{306\text{-JL}}$	$NBC^{(2)}_{306\text{-JL}}$	Concl.
189	(Case)	0.5065	0.5044	———
356	(Case)	0.5079	0.6837	Improved
361	(Case)	0.5242	0.8171	Improved
758	(Case)	0.5244	0.7517	Improved
1114	(Case)	0.4706	0.9645	Corrected

(J)

Instance No.		$NBC^{(2)}_{306\text{-JL}}$	$NBC^{(1)}_{306\text{-JL}}$	Concl.
110	(Case)	0.5002	0.9121	Improved
189	(Case)	0.5044	0.5065	Improved
789	(Ctrl)	0.4711	0.0376	Improved

(K)

Instance No.		$NBC^{(1)}_{306\text{-AA}}$	$NBC^{(2)}_{306\text{-AA}}$	Concl.
1006	(Ctrl)	0.5111	0.1906	Corrected
1724	(Ctrl)	0.4875	0.0811	Improved
2101	(Case)	0.5097	0.9602	Improved
2149	(Ctrl)	0.4562	0.3912	Improved
2675	(Case)	0.5257	0.8650	Improved
2677	(Case)	0.5367	0.8083	Improved
2689	(Case)	0.5335	0.6234	Improved

(L)

Instance No.		$NBC^{(2)}_{306\text{-AA}}$	$NBC^{(1)}_{306\text{-AA}}$	Concl.
526	(Case)	0.5461	0.7580	Improved
628	(Case)	0.5163	0.9617	Improved
643	(Ctrl)	0.4836	0.1185	Improved
838	(Case)	0.5329	0.7508	Improved
1107	(Case)	0.4596	0.7027	Corrected
1663	(Case)	0.5104	0.9597	Improved
1999	(Ctrl)	0.4803	0.1674	Improved
2381	(Ctrl)	0.4796	0.1233	Improved
2430	(Case)	0.517/	0.8694	Improved

Table S3. Accuracy (%) of NBCs evaluated according to fitting/leave-one-out/10-fold cross-validation(CV)

Criterion	NBC with $X_1, \cdots, X_m$ as features					NBC with $Y_1, \cdots, Y_n$ as features				
	100	200	300	400	500	200	400	600	800	1000
Fitting	89.90	97.10	98.60	99.80	99.80	68.50	75.10	78.40	81.70	83.20
leave-one-out	89.00	96.40	98.40	99.40	99.60	51.30	50.50	52.60	52.00	52.20
10-fold CV	89.00	96.30	98.40	99.40	99.50	52.40	50.60	54.10	51.20	52.40

Table S4. Sensitivity (%) of NBCs evaluated according to fitting/leave-one-out/10-fold CV

Criterion	NBC with $X_1, \cdots, X_m$ as features					NBC with $Y_1, \cdots, Y_n$ as features				
	100	200	300	400	500	200	400	600	800	1000
Fitting	89.34	96.77	98.03	99.82	99.64	69.67	76.58	80.29	83.33	84.38
leave-one-out	88.35	96.06	97.85	99.28	99.46	55.30	54.76	56.45	56.09	56.28
10-fold CV	88.35	95.89	97.85	99.28	99.28	56.26	54.82	57.82	55.48	56.50

Table S5. Specification (%) of NBCs evaluated according to fitting/leave-one-out/10-fold CV

Criterion	NBC with $X_1, \cdots, X_m$ as features					NBC with $Y_1, \cdots, Y_n$ as features				
	100	200	300	400	500	200	400	600	800	1000
Fitting	90.65	97.51	99.32	99.78	100	66.75	73.15	76.06	79.69	81.72
leave-one-out	89.88	96.83	99.09	99.55	99.78	45.20	44.42	46.90	46.28	46.54
10-fold CV	89.88	96.82	99.09	99.55	99.78	46.63	44.50	48.79	45.41	46.81

Table S6. MCC of NBCs evaluated according to fitting/leave-one-out/10-fold CV

Criterion	NBC with $X_1, \cdots, X_m$ as features					NBC with $Y_1, \cdots, Y_n$ as features				
	100	200	300	400	500	200	400	600	800	1000
Fitting	0.7957	0.9414	0.9718	0.9960	0.9960	0.3587	0.4953	0.5633	0.6301	0.6601
leave-one-out	0.7775	0.9272	0.9677	0.9879	0.9919	0.0049	−0.0081	0.0330	0.0235	0.0280
10-fold CV	0.7775	0.9252	0.9677	0.9879	0.9899	0.0285	−0.0067	0.0654	0.0088	0.0329

Table S7. Classification performance of NBCs (Figure 1 continued) (E) Matthews correlation coefficients (MCCs; suggested by one of the referees) of $\mathtt{NBC}^{(1)}_{634}$, $\mathtt{NBC}^{(1)}_{753}$, $\mathtt{NBC}^{(1)}_{147}$, $\mathtt{NBC}^{(1)}_{517}$, $\mathtt{NBC}^{(1)}_{306\text{-}JL}$ and $\mathtt{NBC}^{(1)}_{306\text{-}AA}$ according to leave-one-out and 10-fold cross-validation (in the form of "mean±std"), where the results of 10-fold cross-validation are computed by repeatedly performing 10-fold cross-validation for 10 times; the "all" column is for the ordinary 10-fold cross-validation, the "max" column is for the best fold (out of the 10 folds), and the "min" column is for the worst fold. (F) MCCs of $\mathtt{NBC}^{(2)}_{634}$, $\mathtt{NBC}^{(2)}_{753}$, $\mathtt{NBC}^{(2)}_{147}$, $\mathtt{NBC}^{(2)}_{517}$, $\mathtt{NBC}^{(2)}_{306\text{-}JL}$ and $\mathtt{NBC}^{(2)}_{306\text{-}AA}$. (G) MCCs of random 300-feature NBCs, where the results of leave-one-out are computed by averaging 10 random NBCs for every data set.

E

Data	Leave-one-out	10-fold cross-validation		
		All (mean±std)	Max (mean±std)	Min (mean±std)
phs000634	1.000000	0.994983±0.001950	1.000000±0.000000	0.980946±0.005718
phs000753	0.998255	0.996246±0.001168	1.000000±0.000000	0.987828±0.004462
phs000147	0.998251	0.994581±0.001220	1.000000±0.000000	0.979927±0.007146
phs000517	0.995615	0.995027±0.002092	1.000000±0.000000	0.978241±0.012201
phs000306 (JL)	0.998799	0.994958±0.002455	1.000000±0.000000	0.980814±0.006220
phs000306 (AA)	0.998599	0.996778±0.001370	1.000000±0.000000	0.988833±0.004872

F

Data	Leave-one-out	10-fold cross-validation		
		All (mean±std)	Max (mean±std)	Min (mean±std)
phs000634	0.998997	0.996790±0.001036	1.000000±0.000000	0.988011±0.004155
phs000753	0.998253	0.995636±0.001365	1.000000±0.000000	0.980036±0.007054
phs000147	0.999126	0.996154±0.000940	1.000000±0.000000	0.986937±0.004565
phs000517	0.994140	0.993118±0.001958	1.000000±0.000000	0.975358±0.013682
phs000306 (JL)	0.998800	0.995078±0.002430	1.000000±0.000000	0.978470±0.012427
phs000306 (AA)	0.998600	0.995311±0.001043	1.000000±0.000000	0.986713±0.003979

G

Data	Leave-one-out	10-fold cross-validation		
		All (mean±std)	Max (mean±std)	Min (mean±std)
phs000634	0.719564±0.009181	0.718224±0.007050	0.797215±0.022714	0.634884±0.023491
phs000753	0.701933±0.009999	0.699119±0.009361	0.771391±0.024923	0.637271±0.018032
phs000147	0.691249±0.009005	0.686718±0.009956	0.756283±0.022465	0.606278±0.030169
phs000517	0.289578±0.030874	0.288875±0.032605	0.401884±0.035301	0.180312±0.043995
phs000306 (JL)	0.365867±0.020679	0.366504±0.020973	0.477780±0.046572	0.251098±0.031080
phs000306 (AA)	0.309344±0.011395	0.308562±0.010901	0.382190±0.029963	0.228295±0.024264
